# Community characterization of soil from the Central Deccan Plateau dry tropical deciduous forest (Ceddtrof) in central India using 16S rRNA gene amplicon sequencing

**DOI:** 10.1128/mra.01134-23

**Published:** 2024-01-25

**Authors:** Bhagwan Narayan Rekadwad, Yogesh S. Shouche, Kamlesh Jangid

**Affiliations:** 1 National Centre for Microbial Resource, DBT-National Centre for Cell Science, SP Pune University Campus, Pune, Maharashtra, India; 2 Microbe AI Lab, Division of Microbiology and Biotechnology, Yenepoya Research Centre, Yenepoya (Deemed to be University), Mangalore, Karnataka, India; 3 Gut Microbiology Research Division, SKAN Research Trust, Bangalore, Karnataka, India; 4 Bioenergy Group, DST-Agharkar Research Institute, Pune, Maharashtra, India; DOE Joint Genome Institute, Berkeley, California, USA

**Keywords:** forest soil microbiome, 16S rRNA gene sequencing, next-generation sequencing, MTP microbiome pipeline, planctomycetes, evolutionary microbiology, Chloroflexi, Acidobacteria, host-microbe interaction, microbial dark matter

## Abstract

We report a preliminary study of soil from the Central Deccan Plateau dry tropical deciduous forest in India using 16S rRNA gene amplicon sequencing. We report diverse taxa, e.g., Proteobacteria, Actinobacteria, Acidobacteria, Plactomycetes, Chloroflexi, Bacteroidetes, Verrucomicrobia, Gemmatimonadetes, Firmicutes, Crenarchaeota, Nitrospirae, Armatimonadetes, Elusimicrobia, Cyanobacteria, Chlamydiae, Chlorobi, Parvachaeota, Tenericutes, Euryarchaeota, Fibrobacteres, Calditrix, and Spirochaetes.

## ANNOUNCEMENT

Soil from the Central Deccan Plateau dry tropical deciduous forest (Ceddtrof) in Ceddtrof, northeast Maharashtra, is rich in organic nutrients from the degradation of long leaves of trees, balanced water activity, ionic concentration, intense sunlight, high temperatures, and organic matter decomposing and nurturing the soil microbiome (1–4). Ceddtrof typically contains Actinomycetes (*Streptomyces* spp.) that produce secondary metabolites (5), such as antibiotics (6–8). The knowledge gained from studying these environments can provide insights into the existence of Candidatus taxa (9). A thick soil layer, approximately 12 cm in thickness, was collected five times in a 60-feet by 60-feet plot by the composite sampling method (Table 1).

**TABLE 1:** Metadata and details of features in soil samples collected from tropical deciduous forest^*b*^

Place	Sample ID	Adapters/barcode sequence	Sequence Read Archive (SRA) numbers	Forward sequence count	Reverse sequence count	Coordinates		Temperature (^o^C)	pH	Total number of features^*a*^	Total number of OTUs^*c*^ per sample
						Latitude	Longitude				
Met	KVRF01	AAAAAAAAT	SRR25014913	242,622	242,622	19°38′22.8″	77°55′18.1″	22	6.5	1,249,229	7,207
Kurali	KVRF02	AAAAAAAAC	SRR25014914	231,244	231,244	19°38′24.5″	77°58′10.7″	22	7.0		
Ghamapur	KVRF03	AAAAAAATT	SRR25014915	226,647	226,647	19°38′22.3″	78°58′10.6″	23	7.0		
Rampur	KVRF04	AAAAAAATC	SRR25014916	211,634	211,634	19°37′44.2″	78°00′02.9″	23	7.0		
Korta	KVRF05	AAAAAAAGT	SRR25014917	209,622	209,622	19°37′47.0″	78°03′72.7″	24	7.5		
Pandhra Phata	KVRF06	AAAAAAAAG	SRR25014918	127,577	127,577	19°38′27.9″	78°04′39.8″	26	7.5		

^*a*^ A total of 1,249,229 features (reads) were detected after filtering out low-quality, truncation, nontarget, and chimeric amplicons and removing ambiguous bases, and were used for taxonomic analysis and phylogeny.

^*b*^ In the table.qzv QIIME 2 artifact, and in particular the Interactive Sample Detail tab in that visualization. Sampling depth is the value chosen to pass for --p-sampling-depth. Here we set the --p-sampling-depth parameter to 89,784. This value was chosen based on the number of sequences in the sample based on feature table (https://docs.qiime2.org/2023.9/tutorials/moving-pictures/) (10).

^*c*^ OTU, operational taxonomic unit.

**Fig 1:**
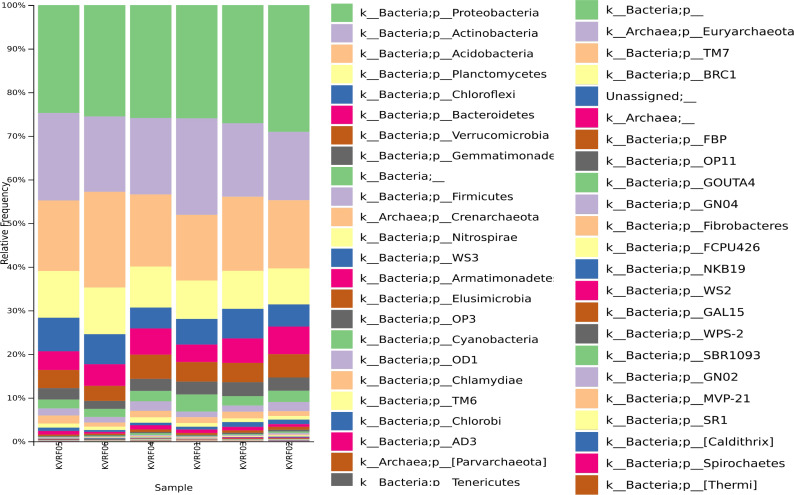
Community characterization of the Ceddtrof, India. Taxonomic bar plot indicating the relative abundance of different phyla.

The upper 2-cm humus layer containing organic matter was discarded, and the remaining 10-cm soil layer was saved. Soil was collected five times to make one composite sample for each site. Six composite samples were collected, labeled as KVRF01–KVRF06, and separately packed in Nasco Whirl-Pak sampling bags (PW390, HiMedia Laboratories) (11, 12). All the samples were subsequently transported to the laboratory on dry ice. The environmental temperature and pH of the samples recorded at the sampling site were ~23°C and 6.5–7.5, respectively. The maximum temperature recorded during transportation was 5°C. DNA was extracted from the composite soil samples using a QIAGEN DNeasy PowerSoil Pro Kit 50 Prep (Cat. No. 47014) according to the manufacturer’s protocol (13). Locus-specific sequence primers (forward 5′GTGCCAGCMGCCGCGGTAA3′) and (reverse 5′GGACTACHVGGGTWTCTAAT3′), were used to amplify the V4 region of the bacterial 16S rRNA gene (14, 15). PCR amplicon libraries were prepared using Nextera XT DNA Library preparation kit (Illumina, Inc., USA) following the manufacturer’s instructions (15, 16). To obtain final libraries, final clean-up was conducted using AMPure XP beads, which were then examined for fragment size distribution using TapeStation (5067–5582, Agilent Technologies) and quantified using Qubit DNA (Q32854, Thermo Fisher Scientific) prior to sequencing. We used the Illumina MiSeq platform with paired-end 2 × 250 bp chemistry to sequence the 16S rRNA gene amplicon libraries. Taxonomic profiling was performed for feature, statistical analysis, differential abundance, and diversity analysis using the stand-alone QIIME2 version 2023.9 (10). A total of 7,207 operational taxonomic units were identified and belonged to 17 phyla (Table 1), such as Proteobacteria, Actinobacteria, Acidobacteria, Plactomycetes, Chloroflexi, Bacteroidetes, Verrucomicrobia, Gemmatimonadetes, Firmicutes, Crenarchaeota, Nitrospirae, Armatimonadetes, Elusimicrobia, Cyanobacteria, Chlamydiae, Chlorobi, and some other phyla, such as Parvachaeota and Tenericutes, Euryarchaeota, Fibrobacteres, Calditrix, and Spirochaetes were also detected. The remaining taxa <3% belonged to more rare taxa such as WS3, OP3, OD1, TM6, AD3, TM7, BRC1, FBP, OP11, GOUTA4, GN04, FCPU426, NKB19, WS2, GAL15, WPS-2, SBR1093, GN02, MVP-21, and SR1 (Fig. 1).

## Data Availability

The 16S rRNA gene amplicon sequence data from this study have been deposited in GenBank SRA under accession numbers SRR25014913–SRR25014918 and BioProject accession number PRJNA987149.
